# The Combination of Iron and Copper Increases Pathogenicity and Induces Proteins Related to the Main Virulence Factors in Clinical Isolates of *Cryptococcus neoformans* var. *grubii*

**DOI:** 10.3390/jof8010057

**Published:** 2022-01-06

**Authors:** Nórida Vélez, Lucía Monteoliva, Zilpa-Adriana Sánchez-Quitian, Ahinara Amador-García, Rocío García-Rodas, Andrés Ceballos-Garzón, Concha Gil, Patricia Escandón, Óscar Zaragoza, Claudia-Marcela Parra-Giraldo

**Affiliations:** 1 Unidad de Proteómica y Micosis Humanas, Grupo de Enfermedades Infecciosas, Departamento de Microbiología, Facultad de Ciencias, Pontificia Universidad Javeriana, Bogotá 110231, Colombia; velez.norida@javeriana.edu.co (N.V.); adrbiology@gmail.com (Z.-A.S.-Q.); c-ceballos@javeriana.edu.co (A.C.-G.); 2 Departamento de Microbiología y Parasitología, Facultad de Farmacia, Universidad Complutense de Madrid, 28040 Madrid, Spain; luciamon@ucm.es (L.M.); ahinaram@ucm.es (A.A.-G.); conchagil@ucm.es (C.G.); 3 Mycology Reference Laboratory, National Centre for Microbiology, Instituto de Salud Carlos III, Carretera Majadahonda-Pozuelo, 28013 Madrid, Spain; rocio.rodas@gmail.com (R.G.-R.); ozaragoza@isciii.es (Ó.Z.); 4 Department of Parasitology and Medical Mycology, Faculty of Pharmacy, University of Nantes, 44200 Nantes, France; 5 Grupo de Microbiología, Instituto Nacional de Salud, Bogotá 111321, Colombia; pescandon@ins.gov.co

**Keywords:** *Cryptococcus neoformans*, metals, iron, copper, pathogenicity, virulence, proteins

## Abstract

In fungi, metals are associated with the expression of virulence factors. However, it is unclear whether the uptake of metals affects their pathogenicity. This study aimed to evaluate the effect of iron/copper in modulating pathogenicity and proteomic response in two clinical isolates of *C. neoformans* with high and low pathogenicity. Methods: In both isolates, the effect of 50 µM iron and 500 µM copper on pathogenicity, capsule induction, and melanin production was evaluated. We then performed a quantitative proteomic analysis of cytoplasmic extracts exposed to that combination. Finally, the effect on pathogenicity by iron and copper was evaluated in eight additional isolates. Results: In both isolates, the combination of iron and copper increased pathogenicity, capsule size, and melanin production. Regarding proteomic data, proteins with increased levels after iron and copper exposure were related to biological processes such as cell stress, vesicular traffic (Ap1, Vps35), cell wall structure (Och1, Ccr4, Gsk3), melanin biosynthesis (Hem15, Mln2), DNA repair (Chk1), protein transport (Mms2), SUMOylation (Uba2), and mitochondrial transport (Atm1). Increased pathogenicity by exposure to metal combination was also confirmed in 90% of the eight isolates. Conclusions: The combination of these metals enhances pathogenicity and increases the abundance of proteins related to the main virulence factors.

## 1. Introduction

In eukaryotic cells, transition metals (manganese (Mn), iron (Fe), cobalt (Co), nickel (Ni), and copper (Cu)) are involved in redox reactions and are necessary for many important biological processes. In addition, they play a role in infection processes in different fungi and serve as cofactors for several enzymes related to the expression of virulence factors, such as superoxide dismutase (*SOD*), metalloproteases, and preceding laccases [1,2]. In the host, metals are related to the activation and regulation of the immune system [3,4].

Thus, success in establishing the infection depends on the ability of the fungus to compete and acquire metals from the host environment. Pathogenic fungi have developed mechanisms to balance the fine line between essential metal acquisition and defense against their toxicity [1,3]. In the environmental yeast, *Cryptococcus neoformans*, iron and copper regulate the expression of virulence factors and various intracellular signaling pathways, especially those involved in adaptation to environmental changes and stress conditions [5,6].

This fungal pathogen has a polysaccharide capsule that is required for virulence and confers resistance to some stress factors. This structure is anchored to the cell wall, which is composed of glycans, chitin, and glycoproteins [7]. The size of the capsule is variable and depends on the genetic background of the strains and on the environmental conditions. For example, some factors, such as CO_2_, high pH, serum, and low iron and high copper concentrations, lead to an increase in capsule size. Iron and copper have been associated with capsule biosynthesis through the transcription factors, Cuf1 and Cir1 [8,9]. In addition to the capsule, *C. neoformans* accumulates melanin on the cell wall when diphenol compounds such as L-DOPA are present. Synthesis of this pigment occurs through the action of diphenol oxidase (laccase), which has four copper ions as cofactors. Melanin synthesis is regulated by iron and copper. Genes involved in the transcriptional regulation of iron and copper are Cir1 and Cuf1, and their transcription also depends on the activity of siderophores [10,11].

The biological uptake of iron in *C. neoformans* begins at the cell membrane and is mediated by specific ferric reductases (Fre1–4) depending on the metal source (iron salts, ferric citrate, or siderophore–iron complex) [12]. These iron reduction reactions are part of the four mechanisms that are known for the uptake of this metal: (1) the expression of reductase proteins as Cfo1 and Cft1; (2) synthesis and secretion of siderophores as Sit1; (3) enzymes with hemoxygenase activity as Hmx1; (4) acidification of the medium by reducing and non-reducing pathways, such as Fre2 and Cig1 [13,14].

On the other hand, some transporters have been described to be required for the uptake of copper, such as Ctr1 and Ctr4 in the membrane, and the Ccc2 and Ctr2 in the cytoplasm, allowing delivery through ATP hydrolysis [15]. Additionally, in the presence of high levels of copper, the yeasts are capable of activating detoxification mechanisms mediated by Cmt1 and Cmt2 membrane proteins [16,17]. Cuf1 is a transcription factor that activates genes encoding proteins related to the uptake and detoxification of this metal. Furthermore, high copper levels correlate with an increase in capsule size, melanin formation, detoxification of reactive oxygen species (ROS), and cellular respiration [15,18].

*C. neoformans* is the most frequent etiological agent of cryptococcosis in patients with HIV/AIDS [19]. Antiretroviral therapy has adverse effects; one of these is the pathophysiological condition of anemia, which may be due to copper or iron deficiency and for which supplements are prescribed in many cases. Unfortunately, these supplements may benefit the pathogenic fungal infection processes. For mycoses in general, alternative therapies have been proposed, where the regulation of metals in the tissue environment during the establishment of mycosis could be a mechanism for controlling the fungus growth [20,21]. The study of protein expression in pathogenic microorganisms makes it possible to know the magnitude of protein expression and have a global idea of their proteome. In the present investigation, a proteomic approach with a focus on the differential expression of proteins due to the presence of iron and copper was adopted [22].

We evaluated the effect of iron and copper on pathogenicity, capsular size, and melanin production in clinical isolates of *C. neoformans.* In addition, the changes induced by the combination of iron and copper were evaluated by label-free proteomics. Pre-incubation with iron and copper alone or combined increased the virulence in most of the strains, which is associated with enlarged capsules and increased melanin pigmentation. Using proteomics, we found that pre-incubation of *C. neoformans* with both 50 µM iron and 500 µM copper resulted in an increase in the abundance of proteins related to oxidative stress response, cell wall integrity, vesicular traffic, capsule, and melanin synthesis. Moreover, we were able to reproduce these phenotype changes, increasing pathogenicity in eight additional clinical isolates.

## 2. Materials and Methods

### 2.1. Microorganisms

Two clinical isolates of *C. neoformans* var. *grubii* (H0058-I-2807 sequence type (ST) 23 and H0058-I-3102 ST 377) VNI molecular type, recovered from the cerebrospinal fluid of HIV/AIDS positive male patients from the department of Antioquia in Colombia, were used. In these clinical isolates, the effect of iron and copper on the capsule, melanin production, and proteomic response was studied. Isolates, 2807 and 3102, were selected by their pathogenicity profile, previously determined in the *G. mellonella* model (low and high pathogenicity, respectively).

The reference strain, *C. neoformans* var. *grubii* H99 ST 2, was used in the phenotypic assays. In addition, eight clinical isolates of *C. neoformans* var. *grubii* were included to confirm the effect of iron and copper on pathogenicity.

### 2.2. Growth Medium

Each isolate was stored at −80 °C. An activation phase was performed on Sabouraud dextrose agar (SDA) at 35 °C for 48 h. To evaluate the effect of iron and copper in all the assays, the clinical isolates, 2807 and 3102, were pre-incubated in a minimal medium (MM) described by Vartivarin et al. (5 g/L glucose, 5 g/L L-asparagine, 0.25 g/L calcium chloride dihydrate, 0.4 mg/L thiamine, 1.2 mg/L zinc sulfate heptahydrate, 0.01 mg/L magnesium chloride tetrahydrate, 80 mg/L magnesium sulfate heptahydrate, 0.46 mg/L sodium molybdate, and 0.057 mg/L boric acid) [23], supplemented with 5, 50, or 500 µM of iron (FeCl_3_), and 1, 10, 500, or 1000 µM of copper (CuSO_4_) or their combinations, according to each experimental requirement.

### 2.3. Effect of Iron and Copper on the Pathogenicity of C. neoformans in G. mellonella

Killing assays were performed in *G. mellonella,* as described by Mylonakis [24]. Briefly, the strains, H99, 2807, and 3102, were cultured in Sabouraud dextrose broth (SDB) at 35 °C with shaking (110 rpm) for 15 h. Then, 5 mL were collected and centrifuged at 1300 rpm for 15 min. The supernatant was discarded, and the pellet was washed twice with phosphate-buffered saline (PBS) solution. Cell concentration was determined using a Neubauer chamber, and inoculum was adjusted to 1.5 × 10^8^ cells/mL in MM supplemented with iron (5, 50 µM) and copper (1, 10, 500, 1000 µM). The cells were re-incubated at 35 °C with shaking (110 rpm) for 15 h. Finally, cells were washed twice with PBS and adjusted to 1 × 10^7^ cells/mL. Subsequently, 10 µL of this inoculum were injected into the last left proleg of larvae using a 0.5 mL BD syringe. Strains were also inoculated into *G. mellonella* after growing only on SDB, as shown in Figure S1.

A group of 10 larvae was used for each of the controls: absolute (uncleaned, uninoculated), disinfection (cleaned with 70% ethanol), and inoculation (received 10 μL of sterile PBS). The number of dead larvae was recorded daily for 15 days. Three biological replicates were performed. Larvae were obtained from the Scientia Colombia SAS breeding facility (La Unión, Valle, Colombia), at late stages (fifth and sixth), with weights of 250–330 mg and a length of approximately 2 cm.

### 2.4. Capsule Induction

Capsule induction was performed following the protocol described by Zaragoza et al., using a capsule-inducing medium (10% SDB in 50 mM MOPS, pH 7.3) [25], and with strains previously incubated with or without 50 µM iron or 500 µM copper, alone or in combination, in MM at 35 °C for 24 h with shaking (110 rpm). Then, an inoculum was adjusted to 1 × 10^8^ cells/mL prior to plating. The cultures were incubated overnight at 37 °C with shaking (110 rpm). Microscopic preparations were carried out, placing one drop of Indian ink and one drop of isolate on a microscope slide. Microscopic observations were conducted using a Leica DMI 3000B microscope (Leica Microsystems). Images were acquired in bright fields with 40× or 63×; objectives, capsule size, and cell body (delimited by cell wall) were measured in 50 cells using the Fiji software [26]. Capsule volume was defined as the difference between the volume of the whole cell (yeast cell + capsule) and the volume of the cell body (as limited by the cell wall). Cell volume measurements were conducted using the following formula: 4/3πr^3^ [[(width + height)/2 = average diameter]; average diameter/2 = radius]].

### 2.5. Melanin Production

Yeast cells were preincubated with and without iron and copper, alone or combined, in MM, as described above. Then, an inoculum was adjusted to 2 × 10^7^ cells/mL in PBS. Serial 1:10 dilutions were performed and 5 μL from each dilution were spotted in a chemically defined minimal medium (CMM) (15 mM dextrose, 10 mM MgSO_4_, 29.4 mM KHPO_4_ 13 mM glycine, 3 μM thiamine, pH 5.5), supplemented with 1 mM L-DOPA (Sigma–Aldrich, St. Louis, MO, USA) and with 1.5% of agar powder. Plates were incubated at 37 °C, protected from light. Pictures were taken daily for 5 days.

### 2.6. Cytoplasmic Protein Extraction

Clinical isolates, 2807 and 3102, were preincubated in 50 mL of SDB at 35 °C with shaking (110 rpm) for 15 h. An inoculum was adjusted to 1.5 × 10^8^ cells/mL in 600 mL of MM and in MM supplemented with 50 µM iron and 500 µM copper, followed by overnight incubation at 35 °C with shaking (110 rpm). Then, the optical density of each culture was measured at 600 nm in a Bioscreen C system (FP-1100-C; Labsystems Oy, Helsinki, Finland). Cell viability was measured with propidium iodide and visualized by microscopy. The metal-exposed cells and untreated controls were washed twice with PBS and collected for protein extraction. Cell extracts were obtained by centrifugation at 1300 rpm for 20 min at 4 °C, the supernatant was discarded, and the pellet was washed three times with PBS. The obtained pellet was stored at −20 °C for 15 h. Then, the pellet was suspended in lysis buffer (50 mM Tris-HCl pH 7.5, 1 mM EDTA, 150 mM NaCl, 1 mM DTT (dithiothreitol)) plus protease inhibitor PMSF (phenylmethyl sulfonyl fluoride) at 0.5 mM (Fluka) and 1% protease inhibitor mix (PierceTM) and disrupted by centrifugation adding glass beads (0.4–0.6 mm diameter) in a Fast-Prep system (Bio101, Savant), applying 20-s pulses 12 times, with intermediate ice cooling (5 min).

The total extracts were centrifuged for 60 min at 14,000 rpm at 4 °C. The supernatant was recovered, and the protein concentration was determined following the Bradford method. The samples were stored at −80 °C until their processing for proteomic analysis. For each condition and strain, four biological replicates were reserved.

### 2.7. Protein Preparation for Mass Spectrometry and Label-Free Protein Quantification

The desalted protein digest was analyzed by RP-LC-ESI-MS/MS in an EASYnLC1000 System, coupled to the Q-Exactive-HF mass spectrometer through the Nano-Easy spray source (Thermo Scientific, Waltham, MA, USA). Peptide identifications were carried out using the Mascotv.2 search engine through the Protein Discoverer Software. A database search was performed against SwissProt. Mascot Scores were adjusted by a percolator algorithm. The acceptance criteria for protein identification were a false discovery rate (FDR) <1% and at least one peptide identified with high confidence (CI > 95%). To determine the abundances of the identified peptides and proteins, a processing free label workflow was initiated in the first step. Finally, the results were normalized to the total amount of the peptides, equaling the total abundance among the different samples. The proteomics data were deposited to the ProteomeXchange Consortium via the PRIDE [27,28].

### 2.8. Statistical Analysis

For numerical variables, measures of central tendency were applied, and for categorical variables, the Chi-square test or Fisher’s exact test were used; the results were considered statistically significant with *p*-values ≤ 0.05. GraphPad Prism 5 software or Stata software Version 11.1 were used. The capsule results were compared using a one-way analysis of variance (ANOVA) applying Levene’s test of homogeneity of variance and Bonferroni’s test of multiple comparisons. Survival curves were constructed using the method of Kaplan–Meier, then the curves were compared using the log-rank (Mantel–Cox) test. Statistical significances are highlighted with asterisks in the figures, as follows: *p* > 0.05, not significant (ns); *p* ≤ 0.05 and >0.01 (*); *p* ≤ 0.01 and *p* > 0.001 (**); *p* ≤ 0.001 and *p* > 0.0001 (***); *p* ≤ 0.0001 (****).

### 2.9. Biological Data Bases and Bioinformatic Resources

The FungiDB website (https://fungidb.org/fungidb, accessed on 1 March 2021), STRING (https://string-db.org, accessed on 1 March 2021), REVIGO (http://revigo.irb.hr/, accessed on 1 March 2021), and KEGG (https://www.genome.jp/kegg/, accessed on 1 March 2021) were used for protein analysis. Gene ontology (GO) was performed on FungiDB (http://fungidb.org; accessed on 1 February 2021) and was used to search for enriched GO terms in the input list of the identified *C. neoformans* gene; the products were compared with the genes from the *C. neoformans* H99 genome. Terms with a *p*-value < 0.05, corrected with the Benjamini–Hochberg test from a calculated and curated evidence list, were included. Hypothetical proteins found (20–30%) of the total proteins identified were not included in the analysis. A Venn diagram was generated using Jvenn software (http://jvenn.toulouse.inra.fr, accessed on 1 March 2021).

## 3. Results

### 3.1. Evaluation of the Pathogenic Capacity of the H99 Strain in Different Pre-Incubation Concentrations of Iron and Copper

**Figure 1:**
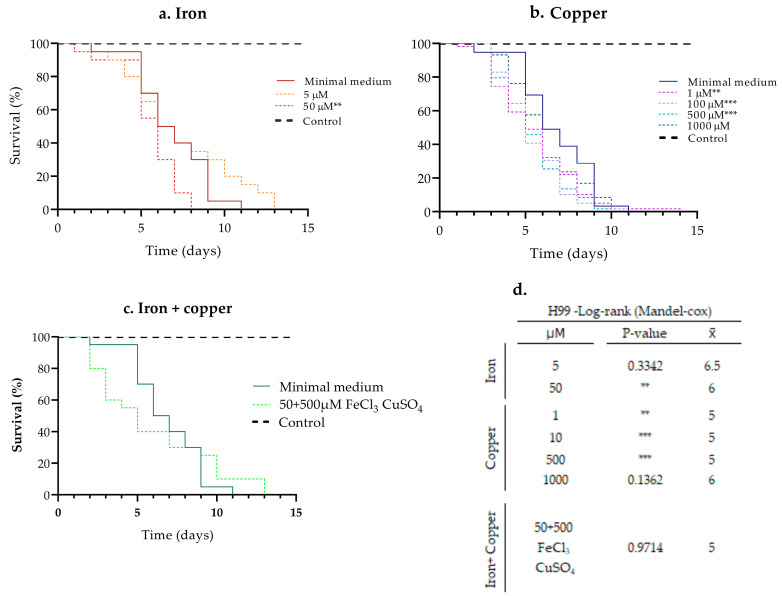
Effect of the pre-incubation with (**a**) iron, (**b**) copper, and (**c**) their combination on the pathogenicity of H99 in *G. mellonella.* (**d**) The table shows the results of the statistical test. *p* ≤ 0.01 (**); *p* ≤ 0.001 (***). The results correspond to three biological replicates; $\bar{x}$: average mortality.

In strain H99, the effect of 5 and 50 µM iron, 1, 10, and 500 µM copper and their combination on pathogenicity was determined using (1.0 × 10^7^ cells/mL) which allowed us to slowly follow the effect of the metals. The 500 µM iron concentration was not used since it significantly affected kinetic yeast growth (Figure S2). The pre-incubation with 50 µM iron significantly increased the pathogenicity of the H99 strain in comparison with the cells grown under the other iron concentrations and without the pre-incubation with iron. In the MM growth condition, H99 caused 100% mortality of larvae by day 11 ($\bar{x}$ = 7), whereas H99 preincubated with 50 µM iron, caused 100% mortality after 8 days ($\bar{x}$ = 5) of follow-up. At a concentration of 5 µM, H99 caused 100% mortality by day 12 ($\bar{x}$ = 7) without significant variation in survival, although a delay in the death of some larvae was observed (Figure 1a,d).

Pre-incubation with 1, 100, and 500 µM copper significantly increased pathogenicity compared to the strain grown without copper. When H99 was grown in MM, yeast caused 100% mortality by day 11 ($\bar{x}$ = 7), whereas H99 preincubated with 1, 100, and 500 µM of copper caused 100% mortality after 14 ($\bar{x}$ = 5), 13 ($\bar{x}$ = 5), and 10 ($\bar{x}$ = 5) days, respectively. At a concentration of 1000 µM, 100% of mortality was observed after 13 days ($\bar{x}$ = 6), showing no significant change in survival percentages compared to growth in MM (Figure 1b–d).

Considering that 50 µM iron and 500 µM copper concentrations induced the most interesting changes in pathogenicity, we then evaluated the effect on pathogenicity using only this combination of metal concentrations. Interestingly, a rapid death of larvae was observed during the first 5 days, which then decreased over time, resulting in 100% mortality by day 13 ($\bar{x}$ = 5). In contrast, the growth of H99 in MM resulted in 100% of mortality of larvae by day 11 ($\bar{x}$ = 7) (Figure 1c,d).

### 3.2. Effect of Pre-Incubation with Iron and Copper on the Pathogenicity of Clinical Isolates

**Figure 2:**
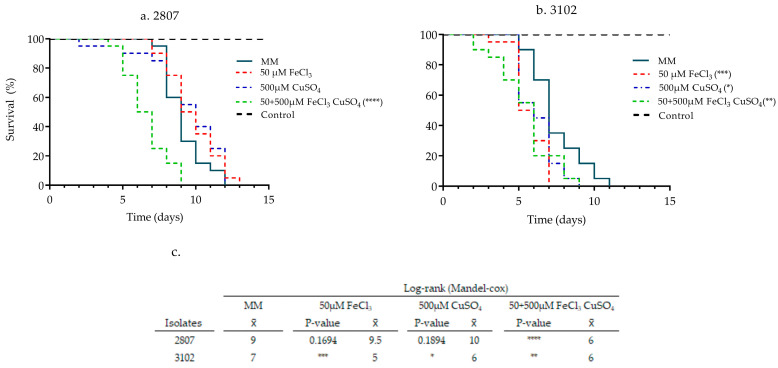
Effect of iron, copper, and their combination on the pathogenicity of two clinical isolates of *C. neoformans*: (**a**) 2807 and (**b**) 3102. (**c**) Results of the statistical survival test for the effect of the pre-incubation with 50 µM iron, 500 µM copper and their combination in *G. mellonella*. The table shows the results of the statistical test; *p* ≤ 0.05 (*); *p* ≤ 0.01 (**); *p* ≤ 0.001 (***); *p* ≤ 0.0001 (****). The results correspond to three biological replicates; $\bar{x}$: average mortality.

An inoculum of 1.5 × 10^8^ cells/mL, as indicated above, was used for isolates 2807 and 3102, with low (100% mortality by day 8 ($\bar{x}$ = 7)) and high (100% mortality by day 5 ($\bar{x}$ = 4)) pathogenicity, respectively (Figure S1). In these isolates, the effect of 50 µM iron, 500 µM copper, and their combination on pathogenicity was determined using a lower inoculum (1.0 × 10^7^ cells/mL). In the MM growth condition, 2807 and 3102 isolates caused 100% mortality of larvae by day 12 ($\bar{x}$ = 9) and 11 ($\bar{x}$ = 7), respectively. Regarding metal use, the pathogenicity of isolate 2807 was only increased after growing it on the iron and copper combination, which resulted in a mortality of 100% of larvae by day 9 ($\bar{x}$ = 6). In contrast, isolate 3102 increased its pathogenicity after pre-incubation with each of the metals, iron 100% by day 7 ($\bar{x}$ = 5), copper 100% by day 9 ($\bar{x}$ = 6), and in combination with 100% mortality by day 9 ($\bar{x}$ = 6) (Figure 2). These results show that the pre-incubation with the iron and copper combination increased the pathogenicity in both isolates.

### 3.3. Effect of Iron, Copper, and Their Combination on Capsular Size

**Figure 3:**
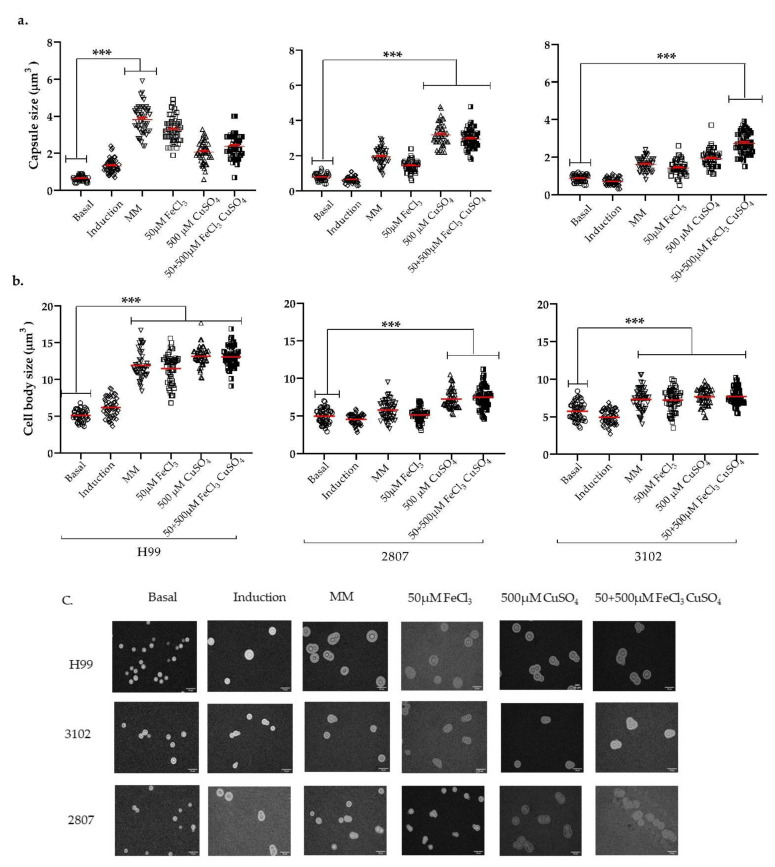
Distribution of the (**a**) capsular size, (**b**) cell size, and (**c**) photographic record of H99, 2807, and 3102. Different conditions were evaluated: basal (cell growth without capsule induction medium and without pre-incubation with metals), capsule induction (without pre-incubation with metals), MM (cell growth in capsule induction plus MM without metals), and 50 µM iron, 500 µM copper, and their combination (growth in capsule induction plus pre-incubation with the different concentrations of metals evaluated). The scale at the bottom right of the images represents 10 µm. *p* ≤ 0.001 (***). The results correspond to three biological replicates.

Since the pre-incubation with 50 µM iron and/or 500 µM copper resulted in a significant change in the pathogenicity of clinical isolates, we next investigated the effect of these metals on different virulence factors. First, we evaluated whether these metals had any effect on capsule size using a capsule-inducing medium. Both H99 and the clinical isolates showed an increase in the capsular size after pre-incubation with metals (Figure 3).

In the H99 strain, the pre-incubation in MM exerts the greatest effect on capsular size, (reaching values of 3.8 µm) compared to the basal capsule size (0.8 µm), followed by iron (3.2 µm), the combination of both metals (2.3 µm), and copper (2 µm) (Figure 3a). Moreover, the average size of the cell body was 5 µm in basal conditions, 7µm in capsule inducing medium, 12 µm in MM, and about 12 µm when metals were used (Figure 3b).

The clinical isolates exhibited a different dynamic in the size of the capsule and cell body compared to the H99 strain. The capsule size in isolate 2807 was larger when 500 µM copper (3.1 µm) was used, compared to the basal condition (0.8 µm). In isolate 3102, the capsule enlargement was greater after metal combination use (2.7 µm), compared to the basal growth condition (0.9 µm) (Figure 3a).

In general, the pre-incubation with copper induced the greatest significant difference in capsule size in the clinical isolates. Remarkably, in the reference strain, H99, which is the most studied strain, a different behavior was observed.

### 3.4. Effect of Iron, Copper, and Their Combination on Melanin Production

We next investigated the effect of metals on melanin production in the L-DOPA medium in the H99 strain and the two clinical isolates. We found that there was an increase in melanin production in strain H99 from day 2 when the metal combination was used, compared to the pre-incubation without metals.

In the clinical isolates, 2807 and 3102, the pre-incubation with 500 µM copper alone and combined accelerated the melanin pigmentation, compared to the basal condition and MM.

**Figure 4:**
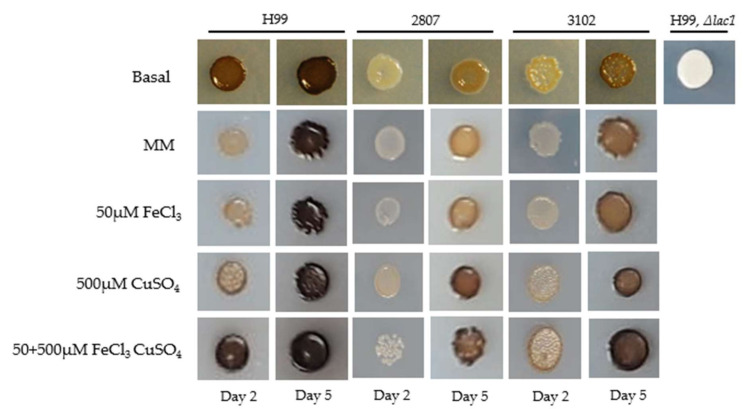
Melanin pigmentation of *C. neoformans* var. *grubii* (H99, 2807, and 3102) in L-DOPA medium. Different conditions were evaluated: SDB, MM (pre-incubation with MM without metals), 50 µM iron, 500 µM copper, and their combination (pre-incubation with the different concentrations of metals evaluated). The experiment was repeated on three different days, obtaining very similar results.

In general, the pre-incubation with copper, alone and combined, in the clinical isolates induced the greatest significant difference in melanin production. Similar results were observed in the reference strain H99. Melanin production was ensured by using a strain lacking the *LAC1* gene (Figure 4).

### 3.5. Proteomic Analysis of the Effect of Iron and Copper

Since the combination of 50 µM iron and 500 µM copper resulted in a modulation of the expression of virulence factors in *C. neoformans*, we decided to gain insights into both the global changes in protein abundance and the changes in proteins involved in the virulence traits. Therefore, protein extraction was performed in the two clinical isolates.

**Figure 5:**
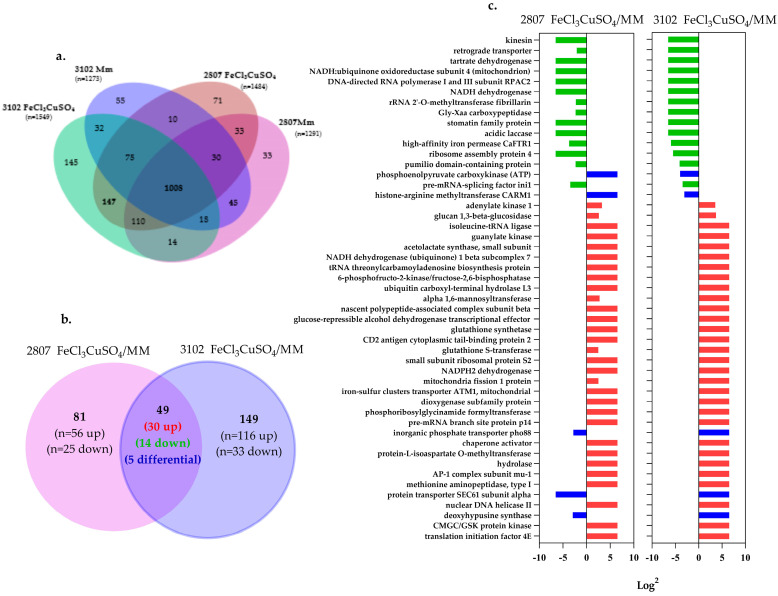
Label-free quantitative proteomics results. (**a**) Venn diagram of total identified proteins in each isolate and condition. (**b**) Venn diagram of the differentially abundant proteins (49 common proteins). (**c**) Proteins that increased and decreased their abundance after metal exposure in both clinical isolates of *C. neoformans* var *grubii.* In red, proteins up-regulated; in green, proteins down-regulated; and in blue, those that presented an opposite abundance between the two isolates.

A total of 1291 and 1484 proteins were identified when isolate 2807 was grown in MM and MM supplemented with metals, respectively. For isolate 3102, 1273 and 1549 proteins were identified on MM and MM supplemented, respectively. The set of common proteins in the two conditions and the two isolates was 1008 (Figure 5a). Among these proteins, 45 and 147 were identified in both isolates after MM and MM supplemented use, respectively (Table S1). Gene ontology (GO) enrichment analysis, using the total of proteins identified, was performed on FungiDB, resulting in a clear difference in the biological processes induced in each of the conditions studied (Table S2).

In the quantitative approach without labeling or label free, each condition was compared (MM incubation as the control versus incubation with iron and copper) in each isolate. The selection of the proteins with a tendency to change was based on those that presented a variability among the replicates of less than 30%, a *q* value <0.05, and a log2 fold change ≥2. Among the selected proteins, the abundance ratio was between −6.64 and 6.64.

In isolate 2807, after metal exposure, 25 proteins were identified as less abundant (i.e., down-regulated) and 56 proteins were identified as more abundant (i.e., up-regulated). For isolate 3102, 33 proteins were identified as down-regulated and 116 proteins were identified as up-regulated. The common proteins identified in both conditions and for both isolates with significant change were 49: 14 as down-regulated, 30 as up-regulated, and 5 proteins with opposite abundances between each isolate (up or down) (Figure 5b,c) (Table S3 sheet 1).

Concerning the five proteins with opposite abundances, two of them (Carm1 and CNAG_04217) increased in isolate 2807 but decreased in 3102; the other three increased in isolate 3102 but decreased in 2807 (CNAG_04822, Sec61 and Pho88).

**Figure 6:**
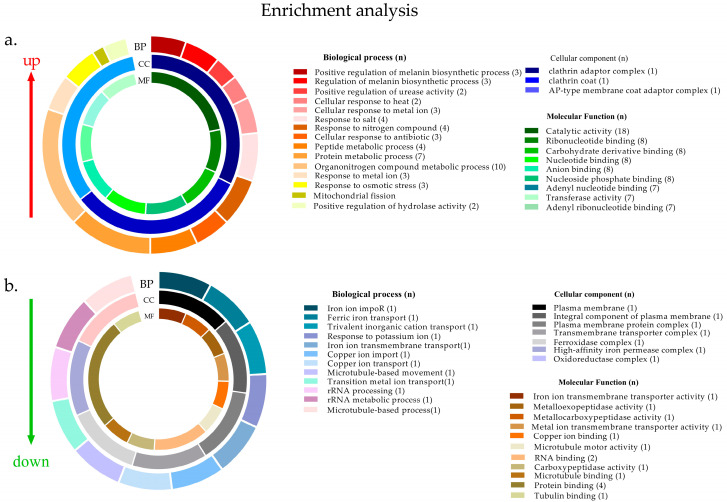
Gene ontology (GO) analysis of the proteins considered differentially abundant after exposure to metals using (**a**) up-regulated (**b**) down-regulated proteins. Number of genes found in each term (n).

Using GO enrichment, several terms were found to be enriched from these 30 up-regulated proteins. Terms were related to the clathrin adaptor complex, melanin biosynthesis, urease or hydrolase activities, heat, salt and metal ion responses, and osmotic stress (Figure 6a and Figure S3). Regarding the 14 down-regulated proteins, terms were related to transmembrane transport activity and response to metal ions, such as the import of ferric iron, copper ion, and potassium (Figure 6b and Figure S4).

We then used the STRING tool to visualize the predicted protein–protein interactions and the KEGG mapping—BlastKOALA database resource for network interaction analysis. The 30 up-regulated proteins were related to purine metabolism, xylene degradation, vernolate III synthesis, photorespiration, fructose synthesis, glutathione biosynthesis, and lipoxin biosynthesis, among others (Figure S3, Table S4). The 14 down-regulated proteins were related to D-malate degradation, and phosphatidylglycerol degradation, among others (Figure S4, Table S4).

### 3.6. Individual Analysis of Proteomic Changes Induced by Iron and Copper in Each Clinical Isolate

An independent analysis of each isolate was then performed. Proteins found with a differential abundance were analyzed (Figure 5b). The GO analysis using the 56 up-regulated proteins in isolate 2807 showed enrichment in terms related to translation processes of the Rho protein signal, translation of the Ras protein signal, the biosynthetic process of coenzyme A, and regulation of biosynthetic processes of lipids, among others (Table S5—sheet 1). The 25 down-regulated proteins were involved in the biotin and NADP biosynthetic process, beta-glucan catabolism, and sexual sporulation, among others (Table S5—sheet 2). The analysis of protein interactions was related to the biosynthesis of L-tyrosine III, proline, coenzyme A, glycolysis and pyruvate pathways, biosynthesis of diacylglycerol, cysteine, trehalose, and biosynthesis of biotin, among others (Table S5—sheet 4).

In isolate 3102, the analysis of the 116 up-regulated proteins showed enrichment in terms related to metabolic processes of UDP-D-xylose, catabolic processes of disaccharides, metabolic synthesis of trehalose, assembly of the spliceosome complex, cellular response to chemical stress, response to oxidative stress, and lipid translocation, among others (Table S5—sheet 3). The 33 down-regulated proteins showed enrichment in terms related to metal cation homeostasis, ion membrane transport, catabolic processes of sulfur compounds, calcium ion transport, TOR signaling, and mitochondrial pyruvate transmembrane transport, among others (Table S5—sheet 4).

The analysis of protein interactions was related to butanoate metabolism, tyrosine biosynthesis, heme I and glycine biosynthesis, aerobic respiration I and II, iron oxidation, L-glutamate biosynthesis, and amino acid assimilation, among others (Table S5—sheet6).

### 3.7. Proteins Related to the Capsule, Melanin, and Other Virulence Factors in Response to the Stimulus-Induced by Iron and Copper Combination

**Figure 7:**
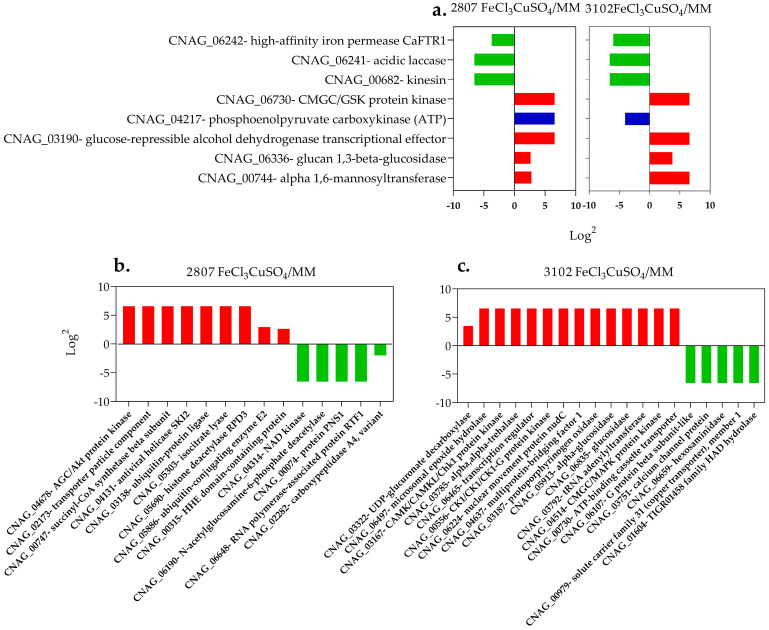
Proteins (*n* = 41) considered differentially abundant related to the capsule, melanin, and other virulence factors (**a**) Proteins common to both isolates. (**b**) exclusive proteins found in 2807 isolate and (**c**) in 3102 isolate. In red, up-regulated proteins; in green, down-regulated proteins; and in blue, those that presented opposite abundances between the two isolates.

From the total of the proteins identified in MM and MM plus metals, 205 are related to capsule biosynthesis, melanin production, and pathogenicity. Some of these proteins are implicated in cytosolic (Ctr2) and endosomal transport (Vps35 protein), capsule biosynthetic regulation (Gsk3 and Chk1), pigment biosynthesis (Mln2 and Hem15), and cell wall synthesis (Och1), among others (Table S6). From the 205 proteins, 41 were found with differential abundances (*q* value <0.05, and a log2 fold change ≥2). As shown in Figure 7a, common proteins (8/41) were identified in both isolates (Och1, Gsk3, Ccr4, CNAG_06336): 3 up- (Cfo1, Cft1 and CNAG_00682-t26_1) and 4 down-regulated, and CNAG_04217 with an opposite regulation. Moreover, 14/41 proteins from isolate 2807: 9 up- and 5 down-regulated. Finally, 19/41 from isolate 3102: 14 up- and 5 down-regulated (Figure 7b–c). Using the up-regulated proteins, enrichment showed processes related to the organization of the outer capsule, iron assimilation, melanin and sphingolipid biosynthesis, lipid regulation, and histone deacetylation, among others. On the other hand, the down-regulated protein enrichment showed processes related to metabolic processes of N-acetylglucosamine, and ion transport, among others (Table S7).

### 3.8. Effect of Iron and Copper Combination on the Pathogenicity of Eight Clinical Isolates of C. neoformans var. grubii in the G. mellonella Model

Given the interesting findings on the pathogenicity when clinical isolates were cultured on iron and copper, we decided to investigate whether the effect of metals in modifying the pathogenicity is maintained in other clinical isolates. Therefore, eight clinical isolates with different levels of pathogenicity (previously determined) were used (data not shown).

**Figure 8:**
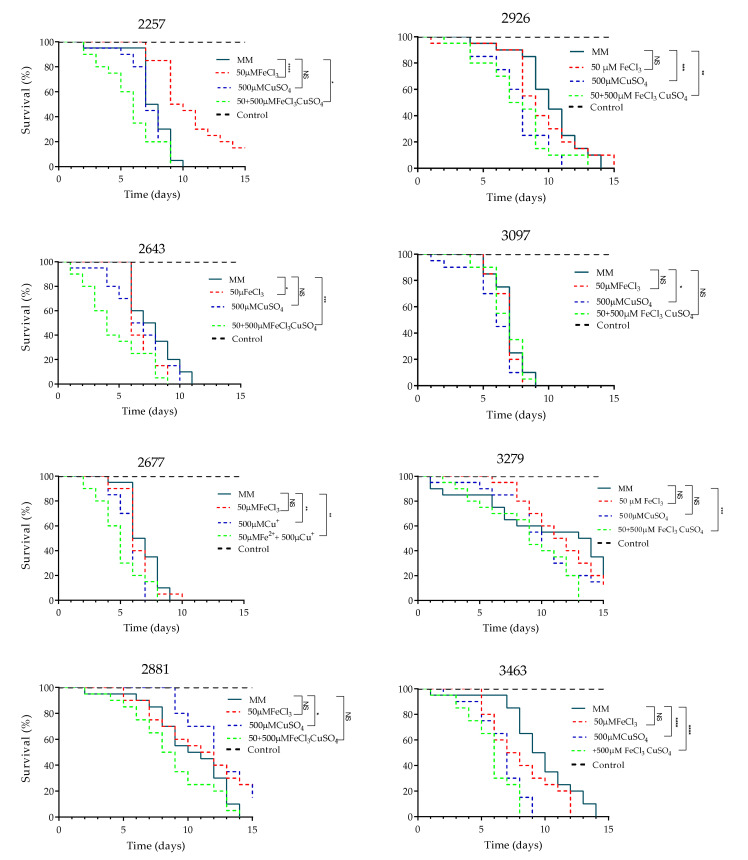
Effect of iron, copper, and their combination on the pathogenicity of eight additional clinical isolates of *C. neoformans* var. *grubii*; *p* ≤ 0.05 (*); *p* ≤ 0.01 (**); *p* ≤ 0.001 (***); *p* > 0.0001 (****).

The isolates showed different pathogenicity profiles; all isolates, except one, caused 100% larval mortality between days 9 and 14 of follow-up. When iron was used, only one isolate enhanced its pathogenicity. However, after exposure to copper alone, and to iron plus copper, 5 out of 8, and 6 out of 8 strains, significantly enhanced their pathogenicity, respectively, showing similar results to those observed in isolates 2807 and 3102 (Figure 8).

## 4. Discussion

Iron and copper are known to play a key role in the modulation of virulence factors in *Cryptococcus* sp. However, their role in the virulence traits of strains with different backgrounds is unknown. This is interesting, since strains may exhibit different phenotypes due to exposure to metals. Furthermore, the evaluation of these metals has usually been carried out independently [29]. Based on this knowledge, we decided to combine iron with copper and to evaluate the effect of the metals, both separately and in combination, on the pathogenicity in *G. mellonella* and on the main virulence traits. We observed, during the infection of *G. mellonella* with yeasts that had been pre-cultured with 50 µM of iron and 500 µM of copper, increased pathogenicity of the strains, mostly when the two metals were used in combination. To our knowledge, this is the first study that addresses the impact of combining iron with copper on *C. neoformans* main virulence factors, pathogenicity in *G. mellonella,* and in a proteomic approach.

Previous studies in *C. neoformans* have shown that pre-incubation with iron or copper affects pathogenicity in murine models and that these metals are involved in the expression of virulence factors [5,23]. It is known that the interruption of iron and copper metalloregulatory factors (*CIR1* and *CUF1*), as well as their permeases, results in pathogenicity alterations [30,31]. For instance, Kronstad et al. described the avirulence of the *cir1* mutant in mice, as well as the loss of capsule production and the alteration of melanin production [14,32]. Thiele et al. determined that strains lacking Cmt proteins or expressing copper-defective Cmt variants exhibit attenuated virulence with little capsule growth and reduced lung colonization [15,33]. In addition, the mutants, *ctr1*, *ctr4*, and *cuf1*, have copper absorption deficiencies and show growth defects under iron conditions, and *ctr1* and *ctr4* reduce the production of melanin in L-DOPA agar [34]. These studies were pivotal in determining that either iron or copper impact growth, melanin synthesis, and capsule size.

In this study, when comparing the effect of metals on each of the virulence traits, a significant impact on these traits was observed, especially after pre-incubation with copper. However, the highest impact was observed with the two metals combined.

Although protein extraction after exposure to iron and copper combined was carried out, it was not possible to detect the metallothioneins, Cmt1 and Cmt2; therefore, it is not possible to know whether they increase or decrease in the presence of the copper concentrations used. A possible explanation for the fact that these proteins were not detected in our study is that they are anchored to the cell wall, and the extraction methodology used does not favor the detection of this type of protein.

Concerning the proteins with increased abundance after exposure to metal combination in both isolates, they are related to vesicular traffic (Ap-1, Vps35), cell wall structure (Och1, Ccr4, Gsk3), pigment biosynthesis (Hem15, Mln2), DNA repair (Chk1), protein transport (Mms2), SUMOylation (Uba2), and a mitochondrial transporter (Atm1), among others. Therefore, the presence of metals in the clinical strains apparently influences the expression of proteins key to pathogenicity.

Regarding the vesicular traffic, the adaptin Ap-1 is an important component of the vesicles that transport ligand-receptor complexes from the plasma membrane or from the trans-Golgi network to the lysosomes. Vps35 is part of the selective cargo complex conformed by Vps26p, Vps29, and Vps35. The latter is the most important central component of the complex mediator of endosome recovery to trans Golgi in yeast [35,36]. Norwood et al. described that Vps35 deletion in H99 confirms that it is a critical component for the cargo recognition complex [36]. Although the association between iron and copper with increased vesicular traffic has not been elucidated, our study suggests that an increased vesicular trafficking may be associated with exposure to these metals.

Among the proteins related to cell wall synthesis, the α-1,6-mannosyltransferase Och1 (involved in the external chain biosynthesis of N-glycans) has been implicated in the pathogenicity of *C. neoformans* because it induces structural changes in the cell wall and affects the exposure of molecular patterns associated with pathogens [37,38]. The Ccr4-Not complex is a conserved eukaryotic regulator; in the genus *Candida*, the disruption of this protein affects the cell wall integrity, susceptibility to antifungal drugs, and adaptation to host temperature [39]. Another protein related to cell wall is the glycogen synthase kinase-3 (Gsk3). In *C. neoformans*, Gsk3 is involved in the regulation of sterols, oxygen detection, sensitivity to CoCl_2_, and cell wall structure [39,40,41]. Both Ccr4 and Gsk3 could be studied as potential drug targets, since Ccr4 is specific to fungi (e.g., *Candida*, *Aspergillus*, and *Cryptococcus*) and Gsk3 is a kinase protein key to pathogenicity—traits that make them interesting candidates for drug development [40,41,42].

Regarding proteins related to melanin biosynthesis, Hem15 and Atm1 have been previously detected after iron exposure [43,44]. In fungi, the enzyme, ferrochelatase (Hem15), responds to light-reducing photosensitization and is, therefore, involved in melanin production [43]. The ABC-type Atm1 transporter encodes an ammonium transporter and is directly regulated by Cir1 [44]. At high levels of copper, the expression of the *ATM1* gene is induced and the deletion of this gene results in a growth defect and a decreased pathogenicity in mice [45]. Those findings are consistent with the phenotypes we observed in our study, confirming that iron and copper are involved in the increase of melanin.

Concerning proteins related to DNA repair processes, cell cycle, and differentiation, such as the Chk1 kinase that is involved in response to DNA damage, the deletion of this gene makes cells highly susceptible to DNA damage stress and increases the susceptibility to certain drugs such as amphotericin B [46]. Other proteins found were Uba2 and Mms2. These belong to the heterodimeric complex that activates SUMO, which intervenes in the process of post-translational modification called SUMOylation; these proteins have been implicated with iron uptake [47,48]. These proteins participate in various metabolic processes in eukaryotes, such as ribosome biogenesis, regulation of the type of mating, control of the cell cycle, and post-replication repair pathway, among other responses [49]. Interestingly, in the two clinical isolates studied, these proteins increased their abundance after exposure to metals. However, no studies have been found in which they have been strongly related to metals. Therefore, further studies are needed to elucidate the relationship between these proteins and metals.

On the other hand, 14 virulence-related proteins with increased abundance were identified in isolate 2807. Rpd304, Ypk1, and Ptp1 are of interest. The histone deacetylase Rpd304 catalyzes the removal of acetyl groups, leading to chromatin condensation and transcriptional repression. In *C. neoformans*, mutations in the genes encoding histone acetylation/deacetylation proteins cause defects in the capsule size and in growth, and some authors suggest that chromatin remodeling could be involved in the regulation of virulence in this fungus [42,50,51]. The Ypk1 kinase is related to the Tor-dependent signaling cascade and is involved in the synthesis of sphingolipids. It is also involved in maintaining the integrity of the cell wall. Disruption of *YPK1* reduces virulence and affects growth, particularly at high temperatures [52,53]. The protein tyrosine phosphatase, known as Ptp1 (CNAG_06064), is indispensable for the regulation of MAPK, and is inducible by stress [54]. Interestingly, Rpd304, Ypk1, and Ptp1 have been proposed as possible therapeutic targets [51,52,54].

Regarding isolate 3102, 19 virulence-related proteins with increased abundance were identified. Mpk1, Afr1, and Mbf1 are of interest. The Mpk1 protein kinase is responsible for cellular integrity at high temperatures. Furthermore, phosphorylation of Mpk1 has been induced in response to disturbances of cell wall biosynthesis by antifungal drugs. Mutant-lacking Mpk1 attenuates its virulence in murine models [55]. MAP kinase and calcineurin pathways control cell wall integrity and promote cell wall remodeling under stress conditions [56]. The ATP-binding protein, Afr1 in *C. neoformans,* is involved in in vitro resistance to fluconazole and virulence [57]. Recent research provided the first evidence that regulation of the *AFR1* gene affects the interaction of *C. neoformans* with the microglia [58]. Finally, the transcriptional coactivator, Mbf1, one of the proteins required for melanin production, was identified. The Mbf1 mutant delays melanization and shows growth and mating defects. Walton et al. proposed that in *C. neoformans,* Mbf1 and Snf5 can act together to regulate *LAC1* expression, inducing melanin production [11].

As explained above, when strains were cultured with iron and copper, capsule-, melanin-, cell wall-, and vesicular traffic-related proteins were detected that could potentially explain the increased pathogenicity observed. To assess whether the phenotype observed in isolates 2807 and 3102 was reproducible in other clinical isolates, we used 8 additional isolates, in which we demonstrated that the combination of iron and copper could trigger pathogenicity increase. This could be a generalized behavior in *Cryptococcus neoformans* var. *grubii* molecular type VNI.

On the other hand, it is now known that the environment has an impact on the virulence traits of pathogens [59]. For instance, several studies with *Cryptococcus* spp. have proposed that the interaction with environmental predators such as amoebae has led to a selective pressure on this yeast that has resulted in the evolution of traits that allow it to be prepared for extreme environments, and to have a better adaptation, i.e., to have an advantage during infection in mammals [60]. Similarly, our results show that metals could provide a higher pathogenic capacity to fungi so that when the pathogen encounters a susceptible host, plus the availability of metals (e.g., HIV/AIDS anemic patients with iron/copper supplementation), they will exacerbate the disease consequently. However, to better understand this process (metals-virulence) and its molecular mechanisms in detail, it is necessary to further explore the dynamics by which metals increase yeast virulence. It would also be of great interest to include in these investigations other stimuli encountered by yeasts in the different environments in which they interact with the host. In addition, further studies should be conducted with the proteins, Rpd304, Ypk1, Ptp1, Mpk1, and Afr1, to confirm their role as therapeutic targets.

## 5. Conclusions

To our knowledge, this is the first investigation that provides insights into the effects of the combination of iron with copper on the pathogenicity and main virulence factors of *C. neoformans* var. *grubii.*

The response to iron and copper stimuli generates different traits. In strain H99, pre-incubation, especially with iron, induced an increase in capsule size. In addition, the combination of iron and copper accelerated mortality in larvae in the first days of infection. In clinical isolates, pre-incubation with iron and copper induced the greatest impact of change in most of the virulence traits evaluated. The isolates increased their capsule and melanin pigmentation and, therefore, enhanced their pathogenicity. The proteomic changes observed in *C. neoformans* var. *grubii* after iron and copper exposure indicate an increase in proteins related to oxidative stress response, cell wall integrity, vesicular traffic, capsule, and melanin production, which apparently explain the observed phenotypes. Furthermore, the phenotypes were corroborated in other clinical isolates. Our findings deserve further studies to understand the complex role that these metals have in *C. neoformans* virulence in its ecological niches and the human host.

## Data Availability

The mass spectrometry proteomics data have been deposited into the ProteomeXchange Consortium via the PRIDE partner repository with the dataset identifier PXD021687.

## Supplementary Materials

The following supporting information can be downloaded at: https://www.mdpi.com/article/10.3390/jof8010057/s1, Figure S1: (a) survival curve of two clinical isolates of *C. neoformans* after growing on SDB, (b) the table shows the results of the statistical test of the isolates evaluated, Figure S2: Growth curve at different concentrations of (a) iron and (b) copper in strain H99, Figure S3: Interaction analysis of proteins common to both clinical isolates that increased in abundance. Results were obtained in KEEG, STRING, and Fungi DB, Figure S4: Interaction analysis of proteins common to both clinical isolates that decreased in abundance. Results were obtained in KEEG, STRING, and Fungi DB. Table S1. Total proteins identified in isolates, 2807 and 3102, of *C. neoformans* after pre-incubation with minimal medium and MM supplemented with iron and copper combination, Table S2: Gene ontology analysis, Table S3. Proteins found with differential abundances in isolates, 2807 and 3102, of *C. neoformans* after pre-incubation with minimal medium and MM supplemented with iron and copper combination, Table S4. Main metabolic pathways identified, Table S5. Enrichment analysis and main metabolic routes identified in isolates, 2807 and 3102, Table S6. Proteins related to capsule biosynthesis, melanin, and other virulence factors, Table S7. Proteins down- and up-regulated linked to capsule and melanin production.
