# Supplementary figures and images for: The Combination of Iron and Copper Increases Pathogenicity and Induces Proteins Related to the Main Virulence Factors in Clinical Isolates of Cryptococcus neoformans var. grubii
Source: J Fungi (Basel). 2022 Jan 6;8(1):57. doi: 10.3390/jof8010057 (PMC8778102; doi:10.3390/jof8010057)

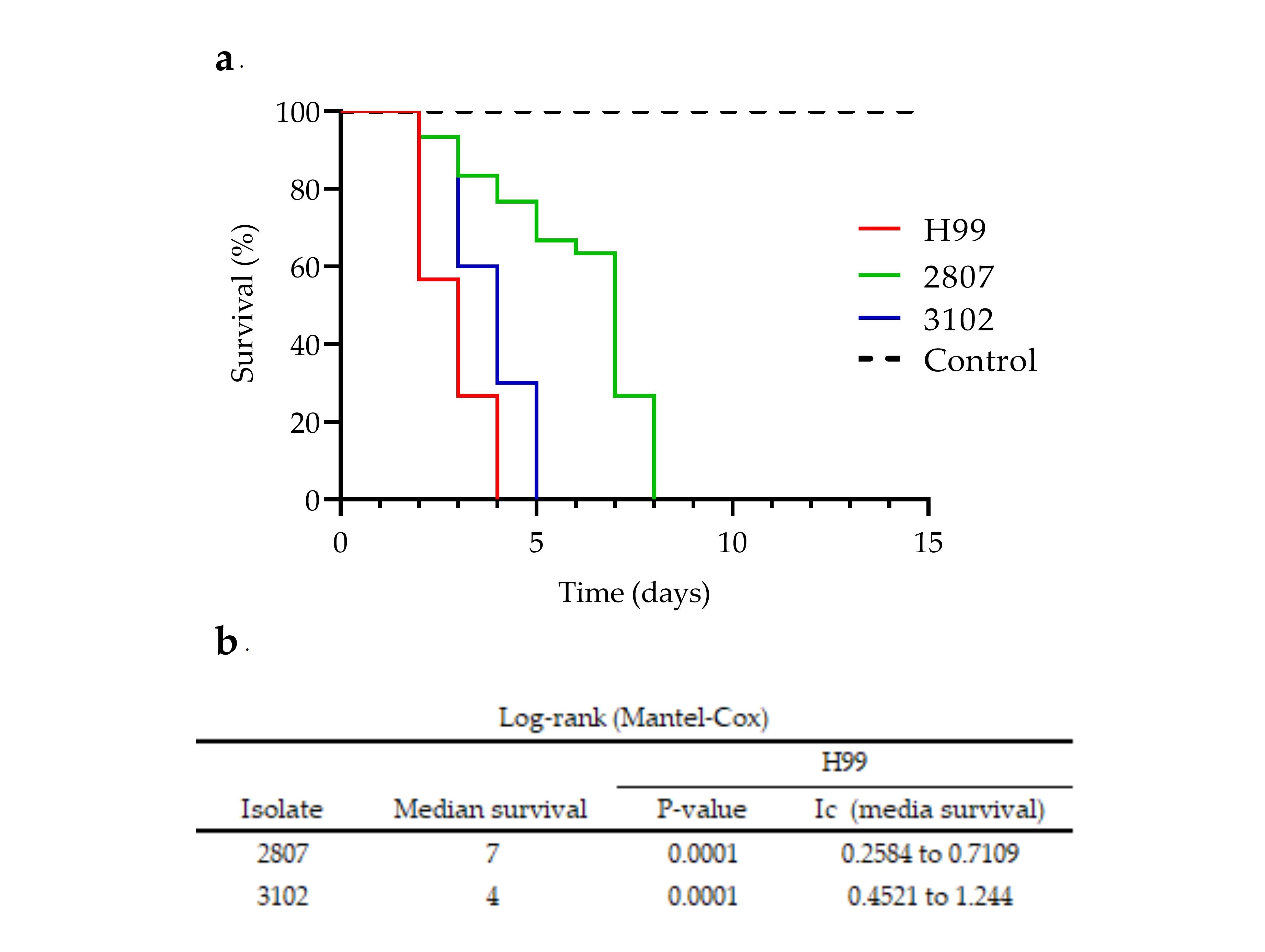

Supplement: Supplementary file 1 [file jof-08-00057-s001.zip › Figure S1.jpg]

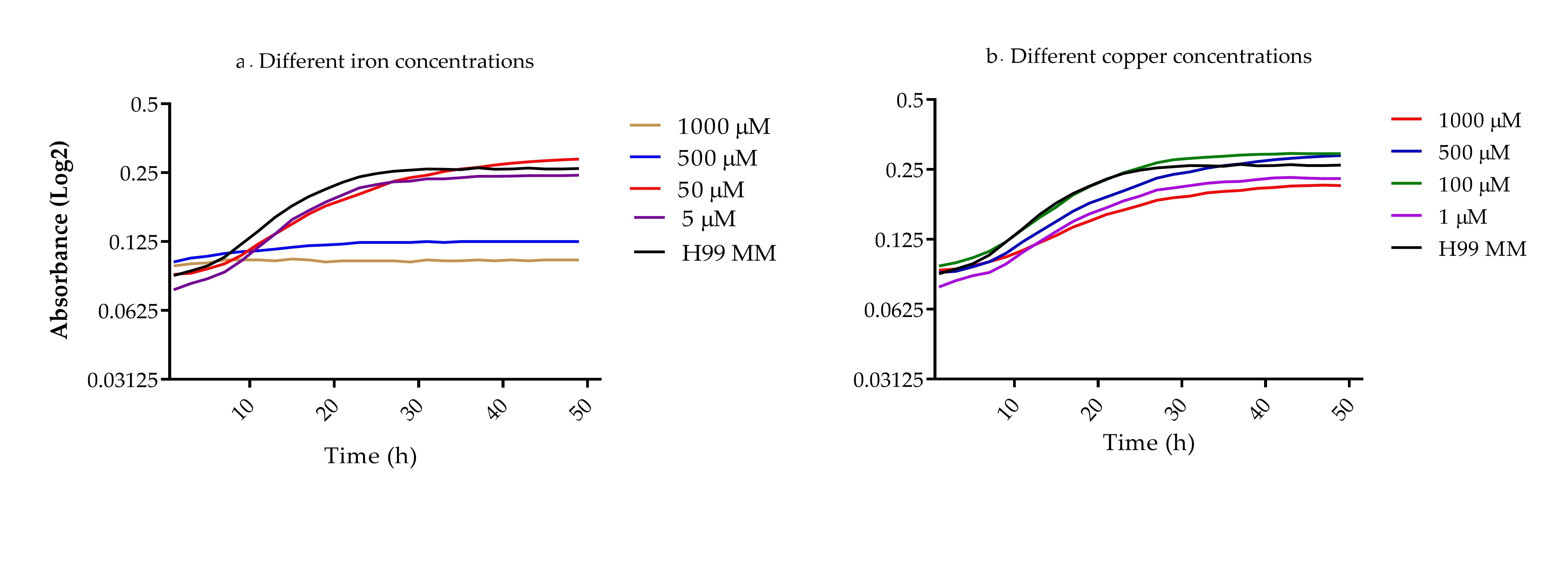

Supplement: Supplementary file 1 [file jof-08-00057-s001.zip › Figure S2.jpg]

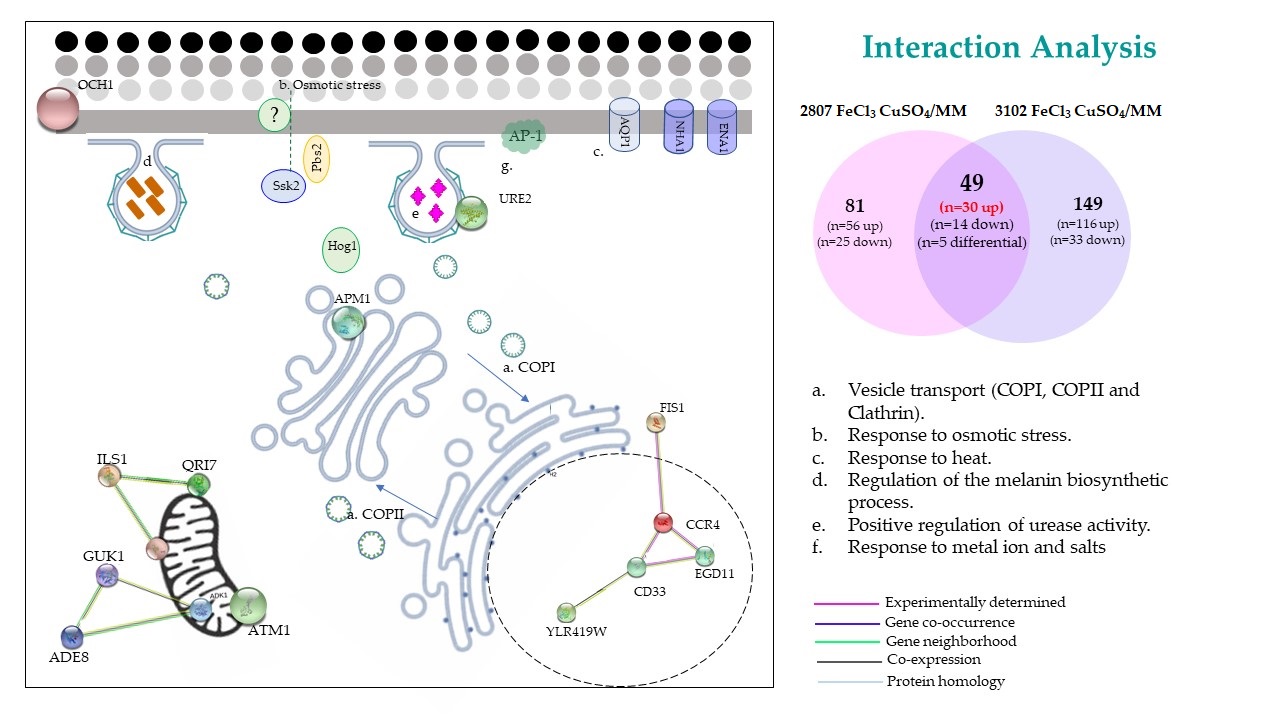

Supplement: Supplementary file 1 [file jof-08-00057-s001.zip › Figure S3.jpg]

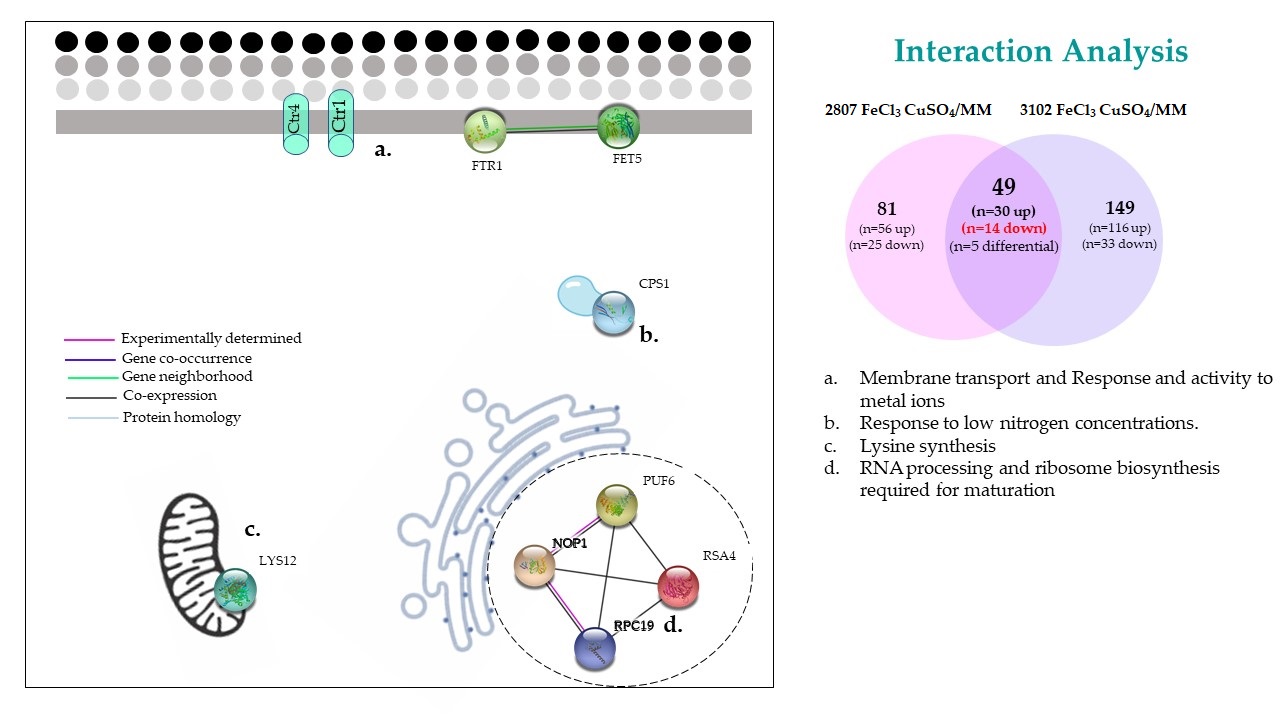

Supplement: Supplementary file 1 [file jof-08-00057-s001.zip › Figure S4.jpg]
